# Generation of a chimeric astrocytic rat spinal cord model by engraftment of human dorsal spinal neural stem/progenitor cells

**DOI:** 10.4103/NRR.NRR-D-24-01176

**Published:** 2025-04-29

**Authors:** Wenjie Xu, Ziyu He, Jia Xu, Ruoying Zhang, Shu Fan, Zhixian Liu, Wei Wang, Hong Chen, Xiaolong Zheng

**Affiliations:** 1 Department of Neurology, Tongji Hospital, Tongji Medical College, Huazhong University of Science and Technology, Wuhan, Hubei Province, China; 2 Hubei Key Laboratory of Neural Injury and Functional Reconstruction, Huazhong University of Science and Technology, Wuhan, Hubei Province, China; 3 Department of Rehabilitation, Tongji Hospital, Tongji Medical College, Huazhong University of Science and Technology, Wuhan, Hubei Province, China; 4 Stem Cell Research Center, Tongji Hospital, Tongji Medical College, Huazhong University of Science and Technology, Wuhan, Hubei Province, China; 5 Key Laboratory of Neurological Diseases of Chinese Ministry of Education, School of Basic Medicine, Tongji Medical College, Huazhong University of Science and Technology, Wuhan, Hubei Province, China

**Keywords:** chimeric, dorsal spinal neural stem/progenitor cells, human embryonic stem cells, human spinal astrocytes, long-term, migration, spinal cord

## Abstract

In the human spinal cord, astrocytes are the major glial cells. *In vitro* studies of human astrocytes are relatively simple. However, the straightforward nature of the *in vitro* environment and complex nature of the *in vivo* environment limit comprehensive investigations into the structure and function of human astrocytes. Additionally, *in vivo* studies of human astrocytes are further limited by ethical issues. This means there is an urgent need to develop effective *in vivo* models to study the structure and function of human astrocytes. Here, we first directed human embryonic stem cells to differentiate into human spinal cord dorsal neural stem/progenitor cells *in vitro*, before transplanting these cells into the gray matter of the cervical spinal cord (C5–T2 segments) of naïve nude rats to create a chimeric human astrocytic rat spinal cord model. The transplanted human spinal cord dorsal neural stem/progenitor cells survived for at least 20 months in the spinal cord environment of the rats, with over 90% differentiating into human astrocytes. These human astrocytes were able to migrate caudally for long distances along the white matter towards the spinal cord. They expressed astrocytic cytoskeletal proteins and functionally-related proteins, suggesting their maturation and structural integration into the rat spinal cord. Thus, this humanized astrocyte chimeric rat spinal cord model provides a valuable tool for studying the role of human spinal cord astrocytes in various spinal diseases.

## Graphical abstract

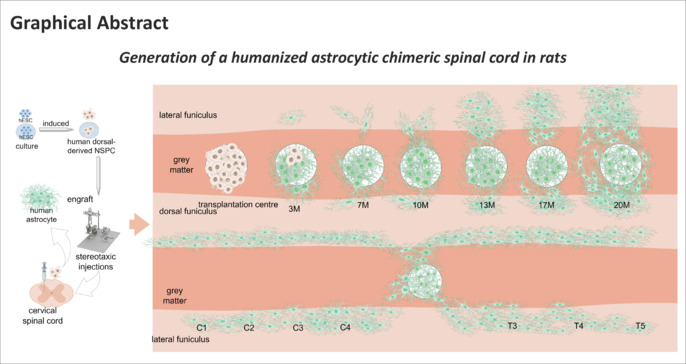

## Introduction

Single-nucleus RNA sequencing has shown that astrocytes constitute more than 10% of the total cells in the adult human spinal cord (Yadav et al., 2023; Zhang et al., 2024). However, it is largely unclear how human spinal astrocytes execute their functions in spinal cord conditions, such as spinal cord injury (SCI), amyotrophic lateral sclerosis, and neuromyelitis optica. While much work has been done on animal models to investigate the functions of spinal astrocytes, the extent to which this evidence can reflect the situation in humans is uncertain. For example, astrocytes become highly active and form dense scars surrounding the lesion core after SCI in rodents (Perez-Gianmarco and Kukley, 2023; Brockie et al., 2024), whereas the astrocytic scar is not prominent in non-human primates, which closely resemble humans (Fan et al., 2023). To date, only a few studies have directly explored the role of spinal astrocytes in SCI (Bunge et al., 1997; Buss et al., 2007, 2009) and amyotrophic lateral sclerosis (Aronica et al., 2001; Catania et al., 2001) in post-mortem human spinal tissue, mainly because it is not possible to examine human spinal astrocytes *in vivo*.

Indeed, studying human astrocytes *in vitro* is comparatively easier. However, the isolation and establishment of primary astrocyte cultures from the human spinal cord is challenging (Whittemore et al., 1994; Palma et al., 1997; Weible and Chan-Ling, 2007). Another more practical and efficient method for the acquisition of human astrocytes is direct differentiation from human embryonic stem cells (hESCs) and human induced pluripotent stem cells (hiPSCs) (Krencik et al., 2011; Krencik and Zhang, 2011; Roybon et al., 2013; Shaltouki et al., 2013; Santos et al., 2017; Tcw et al., 2017; Canals et al., 2018; Li et al., 2018; Bradley et al., 2019; Barbar et al., 2020). Nevertheless, simplified *in vitro* culture conditions lack the complexity of the *in vivo* environment, limiting comprehensive and systematic examination of the structure and function of human astrocytes. Consequently, there is an urgent need to develop effective *in vivo* models to study human spinal astrocytes. In recent years, humanized chimeric rodent brain models have become valuable tools for studying human cerebral neurons (Chen et al., 2016), oligodendrocytes (Wang et al., 2013; Mariani et al., 2019), and microglia (Hasselmann et al., 2019; Xu et al., 2020) *in vivo*. To the best of our knowledge, to date, there has been no humanized spinal neuronal or glial chimeric spinal cord models.

Single-cell RNA sequencing and spatial transcriptomics have shown that during embryonic development, human spinal cord astrocytes are derived from neural stem/progenitor cells (NSPCs) located in the dorsal ventricular zone (Li et al., 2023). Here, we generated human dorsal spinal NSPCs from hESCs and transplanted them into adult naïve nude rat cervical spinal cords to establish an *in vivo* chimeric human spinal astrocytic model.

## Methods

### Cell culture and differentiation

Our previous protocols for the production of spinal motor neurons were modified to differentiate hESCs into dorsal spinal NSPCs (Chen et al., 2014; Du et al., 2015). Briefly, hESCs (H9, WA09, WiCell Research Institute, Madison, WI, USA, Lot No. WB67299, RRID: CVCL_9773) were cultured on irradiated mouse embryonic fibroblasts in six-well plates. The transforming growth factor-β signaling inhibitor SB431542 (2 μM; Stemgent, Beltsville, MD, USA, S1067), bone morphogenetic protein signaling inhibitor DMH1 (2 μM; Selleckchem, Houston, TX, USA, S7146), and glycogen synthase kinase-3 signaling inhibitor CHIR99021 (which activates WNT signaling) (3 μM; Tocris, Minneapolis, MN, USA, 4423/10) were added to the culture medium for 7 days to allow hESCs to differentiate into caudal neuroepithelia. From days 8–14, in addition to the stated inhibitors (SB431542 [2 μM], DMH1 [2 μM], and CHIR99021 [1 μM]), retinoic acid (RA, 0.1 μM; Sigma–Aldrich, St. Louis, MO, USA, R2625) and a Sonic hedgehog signaling antagonist, cyclopamine (0.5 μM; Enzo, Farmingdale, NY, USA, BML-GR334-0001) were further supplemented into the culture medium to direct differentiation of caudal neuroepithelia into dorsal spinal NSPCs. Finally, from days 15–21, SB431542, DMH1, and CHIR99021 were removed from the culture medium, and only RA (0.1 μM) and cyclopamine (0.1 μM) included. These differentiated dorsal spinal NSPCs were further expanded for transplantation.

### Animals

A total of 20 female athymic nude rats, each 6 weeks old (weighing 160–180 g), were obtained from Beijing Vital River Laboratory Animal Technology Co., Ltd. (license No. SCXK [Jing] 2021-0011). A specific pathogen-free environment equipped with a ventilation system was used for animal housing. The temperature within the ventilation cages was maintained between 22°C and 24°C, while the relative humidity was between 45% and 60%. A 12-hour light/dark cycle was used for the animals. Food and water were provided ad libitum on a daily basis, and bedding was changed twice a week. A 2-week acclimation period at the animal facility was provided before commencement of the experiments. All experiments were designed and reported according to the Animal Research: Reporting of *In Vivo* Experiments (ARRIVE) guidelines (Percie du Sert et al., 2020). All experimental procedures were approved by the Committee on the Ethics Committee of Tongji Hospital of Huazhong University of Science and Technology (approval No. TJH-201903007) on March 1, 2019.

### Cell transplantation

Transplantation of NSPCs into the rat cervical spinal cord was conducted as previously described (Lepore, 2011). Anesthesia was administered by intramuscular injection of Zoletil 50 (tiletamine and zolazepam, 10 mg/kg; Virbac S.A., Carros, France) in combination with xylazine (2.5 mg/kg; Sigma-Aldrich, St. Louis, MO, USA). Concurrently, atropine (0.05 mg/kg; Henan Runhong Pharmaceutical Co., Ltd., Xinzheng, China) was used to mitigate the risk of cardiac arrest induced by anesthesia or other factors. Throughout the procedure, a heating pad was used to sustain a body temperature of around 37°C. Additionally, eye ointment was applied bilaterally to prevent xerophthalmia. The hair over the neck was shaved using a razor. The surgical site was disinfected sequentially with iodophor and alcohol, followed by a midline incision. The subcutaneous tissue was bluntly dissected and retracted using a retractor and rongeur to expose the cervical (C)5–C7 spinal segments. The dura mater of the exposed spinal cord was punctured or incised via a needle or scalpel. The rat was then secured in a stereotaxic apparatus (Model 980, David Kopf Instruments, Tujunga, CA, USA) and maintained in the prone position throughout the transplantation procedure. The cells (described above) were resuspended in medium at a concentration of 1 × 10^5^ cells/μL. A glass micropipette was loaded with 2 μL of human dorsal NSPCs and attached to a microinjection pump. The injection site was positioned 1 mm away from the midline and 4 mm beneath the surface of the spinal cord. The cell suspension was administered over a period of 2 minutes, after which the micropipette was left in place for an additional minute before being gradually withdrawn. This procedure was replicated at five additional sites (1 mm away from the midline and 4 mm beneath the surface, 3 points on one side with 1 mm intervals). The connective tissue and skin within the surgical area were closed in layers using interrupted sutures. To prevent dehydration, 5 mL of normal saline was administered subcutaneously. Meloxicam (0.2 mg/kg; Qingdao Vland Biology Co., Ltd., Qingdao, China) and ceftriaxone (50 mg/kg; Shanghai Roche Pharmaceuticals Ltd., Shanghai, China) were administered daily for 3 days post-surgery to facilitate pain relief and prevent infection.

### Tissue processing

At the predetermined observation time points, deep anesthesia was administered by intraperitoneal injection of sodium pentobarbital (60 mg/kg; Fujian Mindong Rejuenation Pharmaceutical Co., Ltd., Ningde, China). A longitudinal incision was made along the abdominal linea alba of the rat, allowing for the separation of tissues and exposure of the heart. The rats were perfused through the left ventricle or aorta with 500 mL of heparinized normal saline, followed by fixation with 500 mL of 4% paraformaldehyde. The brain and spinal cord were carefully dissected in a caudal-to-rostral direction, and collected in 50 mL centrifuge tubes containing identical fixatives. The tissues were fixed at 4°C for 1 day. For the dehydration process, fixative in the centrifuge tube was replaced with a 30% sucrose solution and maintained at 4°C for 72 hours. The C5–thoracic (T)2 segments of the spinal cord were subsequently excised and embedded in embedding substance (Sakura, Osaka, Japan, 4853). This was followed by flash freezing and sectioning into 30 μm horizontal sections. Segments located at the rostral and caudal regions were then sliced into 40 μm thick coronal sections using a cryostat microtome (Leica, Wetzlar, Germany, CM1950). Sections were collected in 24-well plates containing a cryoprotectant solution (30% sucrose and 30% ethylene glycol in phosphate-buffered saline) and stored afterwards at –80°C.

### Immunofluorescence staining

Sections preserved with cryoprotectant and stored at –80°C were brought to room temperature before immunofluorescence analyses. The sections were transferred into 24-well plates, with each well containing 2 mL of Tris-buffered saline with 0.3% Triton X-100. The sections were rinsed three times, each for a duration of 5 minutes, under gentle agitation. The sections were subsequently transferred to a clotting plate and blocked for 15 minutes with Quick Block^TM^ Blocking Buffer (Beyotime, Shanghai, China, P0260). The tissue sections were incubated within a humidified chamber for 16 hours at 4°C with primary antibodies diluted with Quick Block^TM^ primary antibody dilution buffer (Beyotime, P0262). Next, the sections were washed three times with Tris-buffered saline with 0.3% Triton X-100. The secondary antibody solution was incubated with sections in the dark for 1 hour at room temperature. The sections were subsequently rinsed three times, mounted onto slides in Tris-buffered saline with 0.3% Triton X-100, and allowed to dry at room temperature. The slides were labeled and then sealed with Antifade Mountant (Invitrogen, Carlsbad, CA, USA, P36980) at room temperature. Confocal microscopy was utilized to observe the results of immunofluorescence staining and the immunofluorescence images were further processed using Fiji software (National Institutes of Health, Bethesda, MD, USA) (Schindelin et al., 2012). Our findings are illustrated as processed images within the figures. Details of the primary and secondary antibodies are shown in **Tables 1** and **2**.

**Table 1:** Primary antibodies used in the immunofluorescence analysis

	Species	Dilution	Supplier	Catalog number	RRID
NeuN	Chicken	1:1000	Millipore, Burlington, MA, USA	ABN91	AB_11205760
SOX1	Goat	1:200	R&D Systems, Minneapolis, MN, USA	AF3369	AB_2239879
SOX2	Goat	1:200	R&D Systems	AF2018	AB_355110
SOX9	Goat	1:200	R&D Systems	AF3075	AB_2194160
NKX6.1	Goat	1:200	R&D Systems	AF5857	AB_1857045
OTX2	Goat	1:1000	R&D Systems	AF1979	AB_2157172
OLIG2	Goat	1:500	R&D Systems	AF2418	AB_2157554
EAAT2	Guinea pig	1:500	Millipore	AB1783	AB_90949
hNu	Mouse	1:1000	Millipore	MAB1281	AB_94090
HOXC4	Mouse	1:500	OriGene Technologies, Rockville, MD, USA	TA809708	N/A
HOXC9	Mouse	1:200	Abcam, Cambridge, UK	ab50839	AB_880494
SOX2	Mouse	1:500	R&D Systems	MAB2018	AB_358009
PAX3/7	Mouse	1:200	Santa Cruz Biotechnology, Dallas, TX, USA	sc-365843	AB_10842051
nestin	Mouse	1:500	R&D Systems	MAB1259	AB_2251304
hGFAP	Mouse	1:1000	Takara, Kyoto, Japan	Y40420	AB_2833249
NF200	Rabbit	1:500	Sigma‒Aldrich, St. Louis, MO, USA	N4142	AB_477272
CDX2	Rabbit	1:500	Abcam	ab76541	AB_1523334
HOXB8	Rabbit	1:1000	Thermo Fisher Scientific, Waltham, MA, USA	PA5-81200	AB_2788429
FOXG1	Rabbit	1:500	Abcam	ab196868	AB_2892604
Ki67	Rabbit	1:500	Abcam	ab15580	AB_443209
Vimentin	Rabbit	1:500	Abcam	ab92547	AB_10562134
GFAP	Rabbit	1:500	Cell Signaling Technology, Danvers, MA, USA	12389	AB_2631098
S100β	Rabbit	1:500	Abcam	ab52642	AB_882426
ALDH1L1	Rabbit	1:500	Abcam	ab300509	N/A
GLUS	Rabbit	1:500	Abcam	ab176562	AB_2868472
AQP4	Rabbit	1:500	Millipore	AB3594	AB_91530
Cx43	Rabbit	1:500	Abcam	ab235585	AB_3086612
EAAT1	Rabbit	1:500	Abcam	ab181036	AB_2885103
Laminin	Rabbit	1:500	Sigma‒Aldrich	L9393	AB_477163
hNeuN	Rabbit	1:500	Abcam	ab302514	N/A
hMAP-2	Rabbit	1:500	Abcam	ab254263	N/A
hNF-L	Rabbit	1:500	Abcam	ab315814	N/A

ALDH1L1: Aldehyde dehydrogenase 1 family member L1; AQP4: aquaporin 4; CDX2: caudal type homeobox 2; CX43: gap junction protein connexin 43; EAAT1: excitatory amino acid transporter 1; EAAT2: excitatory amino acid transporter 2; FOXG1: forkhead box G1; GFAP: glial fibrillary acidic protein; GLUS: glutamine synthetase; hGFAP: human-specific glial fibrillary acidic protein; hMAP2: human-specific microtube-associated protein 2; hNeuN: human-specific neuronal nuclei; hNF-L: human-specific neurofilament light chain protein; hNu: human nuclei; HOXB8: Homeobox B8; HOXC4: homeobox C4; HOXC9: homeobox C9; NeuN: neuronal nuclei; NF200: neurofilament 200; NKX6.1: NK6 homeobox 1; OLIG2: oligodendrocyte transcription factor 2; OTX2: orthodenticle homeobox 2; PAX3/7: paired box 3/7; SOX1: SRY-box transcription factor 1; SOX2: SRY-box transcription factor 2; SOX9: SRY-box transcription factor 9; S100β: central nervous system specific protein β.

**Table 2:** Secondary antibodies used in the immunofluorescence analysis

	Dilution	Supplier	Catalog number	RRID
Donkey anti-mouse Alexa Fluor 488	1:1000	Invitrogen, Carlsbad, CA, USA	A-21202	AB_141607
Donkey anti-mouse Cy3	1:1000	Jackson ImmunoResearch, West Grove, PA, USA	715-165-150	AB_2340813
Donkey anti-mouse Alexa Fluor 647	1:1000	Invitrogen	A-31571	AB_162542
Donkey anti-rabbit Alexa Fluor 488	1:1000	Invitrogen	A-21206	AB_2535792
Donkey anti-rabbit Cy3	1:1000	Jackson ImmunoResearch	711-165-152	AB_2307443
Donkey anti-goat Alexa Fluor 488	1:1000	Invitrogen	A-11055	AB_2534102
Donkey anti-goat Alexa Fluor 594	1:1000	Invitrogen	A-11058	AB_2534105
Donkey anti-guinea pig Alexa Fluor 488	1:1000	Jackson ImmunoResearch	706-545-148	AB_2340472
Donkey anti-guinea pig Cy3	1:1000	Jackson ImmunoResearch	706-165-148	AB_2340460
Donkey anti-guinea pig Alexa Fluor 647	1:1000	Jackson ImmunoResearch	706-605-148	AB_2340476
Donkey anti-chicken 647	1:1000	Jackson ImmunoResearch	703-605-155	AB_2340379

### Laser scanning confocal microscopy

All immunofluorescence images were scanned and collected using a confocal microscope (Fluor View FV3000, Olympus, Tokyo, Japan). The scanning speed was typically configured to 8 μs/pixel, with the gain, offset, and high voltage consistently set for all channels prior to scanning at 1, 0, and 700, respectively. The laser intensity was carefully adjusted using the Hi–Lo viewing mode to prevent overexposure. To minimize spectral overlap and channel bleed-through, the wavelengths of different channels were strategically set to prevent mutual interference. Additionally, imaging was performed in distinct phases. In Phase 1, Hoechst 33258 (4304–70 nm) and Cy3 (570–580 nm) were captured; in Phase 2, Alexa Fluor 488 (500–540 nm) and Alexa Fluor 594 (610–630 nm) were used; and Alexa Fluor 647 (650–750 nm) was generally placed in Phase 3. To identify regions of interest, the entire field of view was initially surveyed under the eyepiece using a 20× objective lens. Areas with numerous typical positive signals were identified as regions of interest. Once a region of interest was identified, z-stacks were captured using an objective lens with 20× or 60× magnification, and optical zoom ranging from 1 to 5 and resolution of either 800 × 800 pixels or 1024 × 1024 pixels, respectively. To observe the migration of differentiated human astrocytes, a technique for capturing images over time in multiple areas was used to capture entire horizontal or coronal sections of the spinal cord. This was achieved by selecting a matrix area of 3 × 12 or 3 × 3. Each matrix area was imaged with a 10× objective lens and subsequently stitched together to form a complete section. The optical zoom was set at 1 and resolution of 256 × 256 pixels. All images were postprocessed and analyzed using Fiji software.

### Quantification and statistical analysis

GraphPad Prism 10.1.2 (GraphPad Software, Boston, MA, USA, www.graphpad.com) software was used for all statistical analyses. All the data in our study were continuous variables. Data normality was assessed using the Shapiro‒Wilk test, with the results showing that all the variables in our study followed a normal distribution. The data are presented as mean ± standard error of mean (SEM). One-way analysis of variance was performed. If the statistical results found a significant difference among groups, Tukey’s *post hoc* test for multiple comparisons was used to determine the difference. Statistical significance was defined as *P* < 0.05.

## Results

### Generation of human dorsal spinal neural stem/progenitor cells from human embryonic stem cells

**Figure 1:**
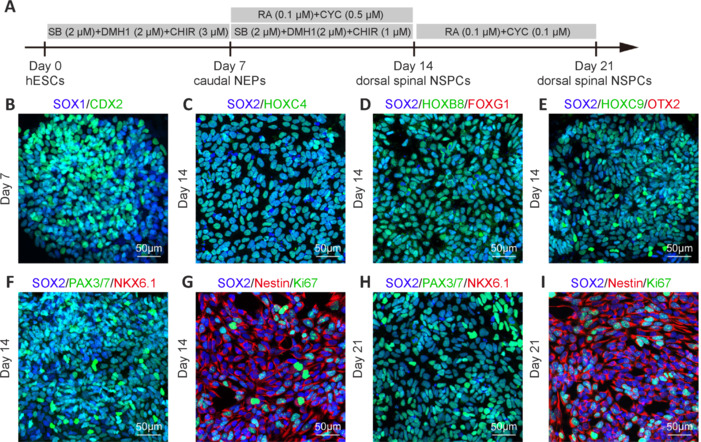
Generation of human dorsal spinal NSPCs from hESCs. (A) Protocol for the differentiation of human dorsal spinal NSPCs from hESCs. (B) On day 7 of neuralization, differentiated cells almost uniformly expressed the neuroepithelia marker SOX1 (blue, Alexa Fluor 594) and the caudal spinal marker CDX2 (green, Alexa Fluor 488). (C–E) On day 14 of differentiation, most SOX2^+^ (blue, Alexa Fluor 594 or Alexa Fluor 647) NSPCs expressed spinal cord-specific markers, including HOXC4 (green, Alexa Fluor 488) (C), HOXB8 (green, Alexa Fluor 488) (D), and HOXC9 (green, Alexa Fluor 488) (E), but not the forebrain marker FOXG1 (red, Cy3) (D) or midbrain marker OTX2 (red, Alexa Fluor 594) (E). (F) On day 14 of differentiation, most SOX2^+^ (blue, Alexa Fluor 647) NSPCs expressed the dorsal spinal markers PAX3 (green, Alexa Fluor 488) and PAX7 (green, Alexa Fluor 488), but not the ventral spinal marker NKX6.1 (red, Alexa Fluor 594). (G) On day 14 of differentiation, SOX2^+^ (blue, Alexa Fluor 594) NSPCs expressed nestin (red, Alexa Fluor 488) and Ki67 (green, Cy3). (H) On day 21 of differentiation, most SOX2^+^ (blue, Alexa Fluor 647) NSPCs expressed the dorsal spinal markers PAX3 (green, Alexa Fluor 488) and PAX7 (green, Alexa Fluor 488) but not the ventral spinal marker NKX6.1 (red, Alexa Fluor 594). (I) On day 21 of differentiation, SOX2^+^ (blue, Alexa Fluor 594) NSPCs expressed nestin (red, Alexa Fluor 488) and Ki67 (green, Cy3). Scale bars: 50 μm in B–I. CDX2: Caudal type homeobox 2; CHIR: CHIR99021, glycogen synthase kinase-3 signaling inhibitor; CYC: cyclopamine; DMH1: bone morphogenetic protein signaling inhibitor; FOXG1: forkhead box G1; hESC: human embryonic stem cell; HOXB8: homeobox B8; HOXC4: homeobox C4; HOXC9: homeobox C9; NEP: neuroepithelia; NKX6.1: NK6 homeobox 1; NSPC: neural stem/progenitor cell; OTX2: orthodenticle homeobox 2; PAX3/7: paired box 3/7; RA: retinoic acid; SB: SB431542, transforming growth factor-β signaling inhibitor; SOX1: SRY-box transcription factor 1; SOX2: SRY-box transcription factor 2.

To convert hESCs into neuroepithelial cells, we used the dual SMAD inhibition protocol (Chambers et al., 2009), which involves the addition of SB431542 and DMH1 (**Figure 1A**). To differentiate neuroepithelia towards a spinal cord phenotype, a glycogen synthase kinase-3 inhibitor (CHIR99021) was added to activate canonical WNT signaling (Chen et al., 2014; Du et al., 2015; Kumamaru et al., 2018; **Figure 1A**). Seven days after neuralization, the cells uniformly expressed SRY-box transcription factor 1 (SOX1), indicating a neuroepithelia identity (**Figure 1B**). Additionally, neuroepithelia evenly expressed the caudal spinal cord marker, caudal-related homoeobox transcription factor 2 (CDX2) (**Figure 1B**). CDX2 activates downstream HOX genes involved in spinal cord patterning (van den Akker et al., 2002). On day 14 of differentiation, SOX2^+^ NSPCs uniformly expressed spinal cord-specific HOX genes, including HOXC4 (**Figure 1C**), HOXB8 (**Figure 1D**), and HOXC9 (**Figure 1E**). In contrast, NSPCs showed no expression of the forebrain and midbrain markers forkhead box G1 (FOXG1) (**Figure 1D**) and orthodenticle homeobox 2 (OTX2) (**Figure 1E**), respectively. Thus, the differentiated NSPCs had a bona fide caudal spinal cord identity. We further directed caudal NSPCs into dorsal spinal NSPCs. Sonic hedgehog signaling is key for ventral patterning of the spinal cord, whereas WNT signaling is integral to dorsal patterning (Rayon et al., 2021). Therefore, cyclopamine as an antagonist of Sonic hedgehog signaling was added to block ventral patterning, while CHIR (which activates WNT signaling) was used to induce dorsal patterning. On day 14 of differentiation, most SOX2^+^ NSPCs expressed the dorsal spinal markers, paired box (PAX)-3 and PAX7, but did not express the ventral spinal marker, NK6 homeobox 1 (NKX6.1) (**Figure 1F**). Furthermore, SOX2^+^ NSPCs were positive for nestin and Ki67 (**Figure 1G**). In continuous presence of RA and cyclopamine, SOX2^+^ cells expanded but maintained their dorsal spinal NSPC identity, and remained positive for PAX3/7 (**Figure 1H**), nestin, and Ki67 (**Figure 1I**) up to day 21. In summary, human spinal dorsal NSPCs were successfully generated from hESCs.

### Differentiation and long-term survival of human astrocytes from grafted human embryonic stem cell-derived dorsal spinal neural stem/progenitor cells in naïve rat cervical spinal cord

**Figure 2:**
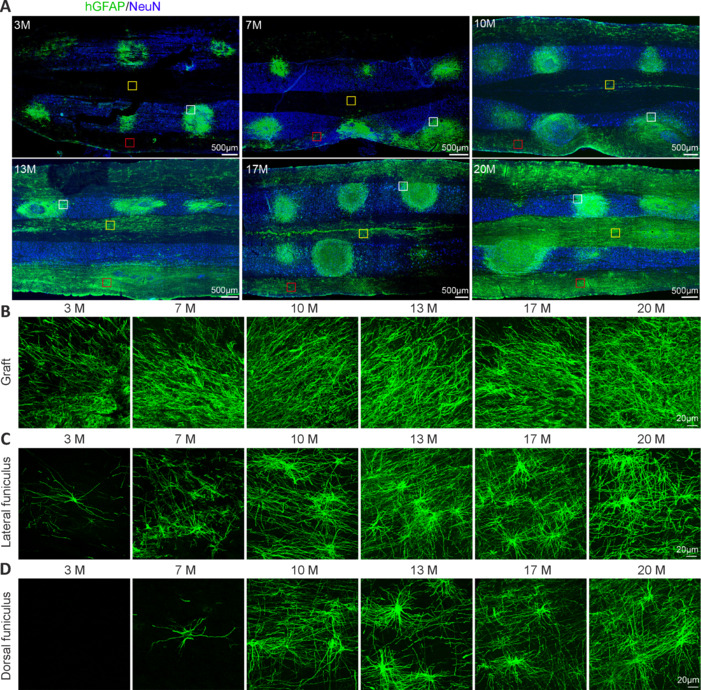
Differentiation and long-term survival of human astrocytes from grafted hESC-derived dorsal spinal NSPCs in the naïve rat cervical spinal cord. (A) Representative images of hGFAP (green, Alexa Fluor 488) and NeuN (blue, Alexa Fluor 647) staining of horizontal sections from C5–T2 segments of the rat spinal cord at 3 to 20 months after transplantation of hESC-derived dorsal spinal NSPCs. (B) Magnified images of the white boxed areas in the graft in A. The hGFAP^+^ cells were grown and migrated from the transplant center. The cells migrated further with increasing time. (C) Magnified images of the red boxed areas in the lateral funiculus in A. Starting at 3 months, hGFAP^+^ cells were found to migrate into the lateral funiculus. Increased hGFAP^+^ cells migrated into the lateral funiculus with increasing time. (D) Magnified images of the yellow boxed areas in the dorsal funiculus in A. Starting at 7 months, hGFAP^+^ cells were found to migrate into the dorsal funiculus. Increased hGFAP^+^ cells migrated into the lateral funiculus with increasing time. Scale bars: 500 μm in A; and 20 μm in B–D. hESC: Human embryonic stem cell; hGFAP: human-specific glial fibrillary acidic protein; M: month; NeuN: neuronal nuclei; NSPC: neural stem/progenitor cell.

Approximately 1.2 million hESC-derived dorsal spinal NSPCs were separately transplanted into six gray matter sites of the cervical spinal cord (C6–C8) of 20 naïve adult female athymic nude rats. One rat died during surgery, possibly due to an overdose of anesthesia. Another rat died nearly 15 months after transplantation. However, the harvested spinal cord could not be used for histology because of its low quality, and this rat was excluded from analysis. The remaining 18 rats were sacrificed 3 to 20 months later. The C5–T2 spinal segments were cut into horizontal sections, followed by staining with human-specific glial fibrillary acidic protein (hGFAP) and neuronal nuclei (NeuN). Survival of hGFAP^+^ human astrocytes within the rat spinal cord gray matter was observed as early as 3 months post-transplantation (**Figure 2A**). These human astrocytes within the graft had either a fibrous or star shape (**Figure 2B**). At 3 months after transplantation, few human astrocytes were present in the rat spinal white matter, including the lateral or dorsal funiculus (**Figure 2C** and **D**). At 7 months after transplantation, an increasing number of human astrocytes were observed in the white matter (**Figure 2C**), especially in the lateral funiculus (**Figure 2C**). During 10–17 months after transplantation, there was extensive migration of human astrocytes into the lateral and dorsal funiculi (**Figure 2C** and **D**). At 20 months after transplantation, the rat spinal white matter, including the lateral (**Figure 2C**) and dorsal funiculus (**Figure 2D**), was fully occupied by numerous human astrocytes. However, extensive migration of human astrocytes along the gray matter was rare at all time points observed (**Figure 2A**). These results suggest that grafted human dorsal spinal NSPCs can differentiate into human astrocytes that survive long-term and migrate extensively within the rat spinal cord.

### Grafted human embryonic stem cell-derived dorsal spinal neural stem/progenitor cells predominantly produce spinal astrocytes within the rat spinal cord

**Figure 3:**
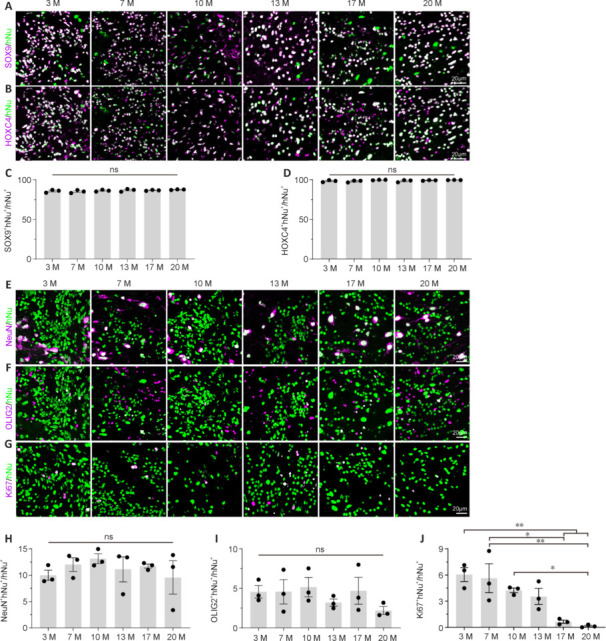
Grafted hESC-derived dorsal spinal NSPCs predominantly produce spinal astrocytes within the rat spinal cord graft. (A, B) Representative images of horizontal SOX9 (magenta, Alexa Fluor 647) (A), HOXC4 (magenta, Alexa Fluor 647) (B), and hNu (green, Alexa Fluor 488) staining of grafts from C5–T2 segments of the rat spinal cord at 3 to 20 months after transplantation of hESC-derived dorsal spinal NSPCs. Numbers of SOX9^+^ cells and HOXC4^+^ cells increased slightly from 3 months (M) to 20 M. (C, D) Quantification of the percentage of SOX9^+^ hNu^+^ (C) and HOXC4^+^ hNu^+^ (D) cells among hNu^+^ cells in grafts over 3–20 months. One-way analysis of variance: SOX9, *F*_(5,12)_ = 1.159, *P* = 0.3833; HOXC4, *F*_(5,12)_ = 1.427, *P* = 0.2832. (E–G) Representative images of horizontal NeuN (magenta, Alexa Fluor 647) (E), OLIG2 (magenta, Alexa Fluor 594) (F), Ki67 (magenta, Cy3) (G), and hNu (green, Alexa Fluor 488) staining of grafts from rat spinal cord C5–T2 segments at 3 to 20 months after transplantation of hESC-derived dorsal spinal NSPCs. Numbers of NeuN^+^ cells and OLIG2^+^ cells showed no evident change from 3 M to 20 M. The number of Ki67^+^ cells decreased from 3 M to 20 M. Scale bars: 20 μm in A, B, E–G. (H–J) Quantification of the percentages of NeuN^+^hNu^+^ (H), OLIG2^+^hNu^+^ (I), and Ki67^+^hNu^+^ (J) cells among hNu^+^ cells in grafts at 3–20 months. One-way analysis of variance: NeuN, *F*_(5,12)_ = 0.5447, *P* = 0.7396; OLIG2, *F*_(5,12)_ = 0.9693, *P* = 0.4741; Ki67, *F*_(5,12)_ = 8.478, *P* = 0.0012. Tukey’s test: Ki67, 3 M *vs.* 17 M, *P* = 0.0076; 3 M *vs.* 20 M, *P* = 0.0038; 7 M *vs*. 17 M, *P* = 0.0137; 7 M *vs*. 19 M, *P* = 0.0069; 10 M *vs.* 20 M, *P* = 0.0477. The data are presented as mean ± SEM. *P < 0.05, **P < 0.01. hESC: Human embryonic stem cell; hNu: human nuclei; HOXC4: homeobox C4; M: month; NeuN: neuronal nuclei; ns: not significant; NSPC: neural stem/progenitor cell; OLIG2: oligodendrocyte transcription factor 2; SOX9: SRY-box transcription factor 9.

To quantify the percentage of differentiated human astrocytes, cells were counted that expressed the astrocyte transcription factor, SOX9, and human-specific cell nuclei (hNu). Indeed, many hNu^+^ human cells within the graft were positive for SOX9 (**Figure 3A**). Additionally, hNu^+^ human cells were uniformly positive for HOXC4 (**Figure 3B**). Quantification revealed that almost 85% of the grafted human cells were SOX9^+^ astrocytes (**Figure 3C**). To further confirm the identity of human astrocytes, the spinal cord-specific marker HOXC4 was examined. Quantification revealed that almost 99% of human cells in the graft were spinal cord cells (**Figure 3D**). In contrast, approximately 10% and 5% of the grafted human cells were NeuN^+^ neurons (**Figure 3E** and **H**) or OLIG2^+^ oligodendroglia (**Figure 3F** and **I**), respectively. In terms of proliferation, approximately 6% of grafted human cells expressed Ki67 at 3 months after transplantation (**Figure 3G** and **J**). This proliferation rate gradually decreased over time to less than 0.1% at 20 months after transplantation (**Figure 3G** and **J**). These results indicate that grafted human dorsal spinal NSPCs produced mostly human spinal astrocytes and minor neurons or oligodendroglia in the rat spinal cord.

### Maturation and structural integration of human spinal astrocytes within grafts of the rat spinal cord

**Figure 4:**
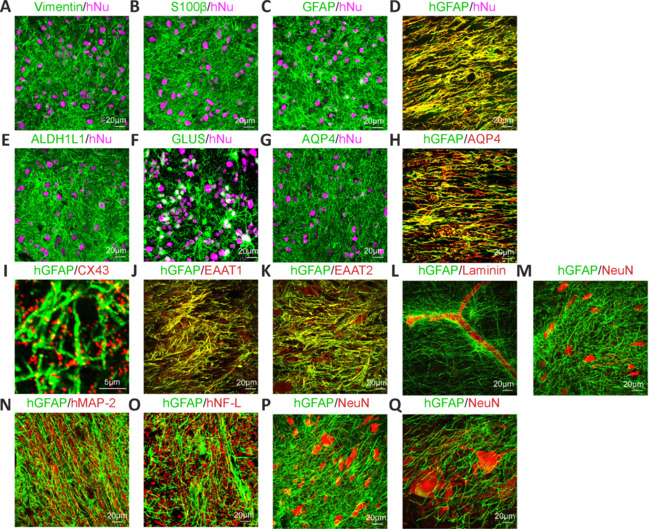
Maturation and structural integration of human spinal astrocytes within grafts in the rat spinal cord. (A–C) Representative images of vimentin (green, Alexa Fluor 488) (A), S100β (green, Alexa Fluor 488) (B), GFAP (green, Alexa Fluor 488) (C), and hNu (magenta, Alexa Fluor 647) staining of grafts in horizontal sections from rat spinal cord C5–T2 segments at 3 months after transplantation. (D) Representative image of GFAP (red, Cy3) (D), and hGFAP (green, Alexa Fluor 488) staining of grafts in horizontal sections from C5–T2 segments of the rat spinal cord at 3 months after transplantation. (E–G) Representative images of ALDH1L1 (green, Alexa Fluor 488) (E), GLUS (green, Alexa Fluor 488) (F), AQP4 (green, Alexa Fluor 488) (G), and hNu (magenta, Alexa Fluor 647) staining of grafts in horizontal sections from rat spinal cord C5–T2 segments at 3 months after transplantation. (H–K) Representative images of AQP4 (red, Cy3) (H), CX43 (red, Cy3) (I), EAAT1 (red, Cy3) (J), and EAAT2 (red, Cy3) (K), and hGFAP (green, Alexa Fluor 488) staining of grafts in horizontal sections from C5–T2 segments of the rat spinal cord at 3 months after transplantation. (L–O) Representative images of laminin (red, Cy3) (L), hNeuN (red, Cy3) (M), hMAP-2 (red, Cy3) (N), hNF-L (red, Cy3) (O), and hGFAP (green, Alexa Fluor 488) staining of grafts in horizontal sections from rat spinal cord C5–T2 segments at 3 months after transplantation. (P, Q) Representative images of NeuN (red, Alexa Fluor 647) and hGFAP (green, Alexa Fluor 488) staining of rat gray matter near grafts in horizontal sections from C5–T2 segments of the rat spinal cord at 3 months after transplantation. Scale bars: 20 μm in A–H, J–Q; and 5 μm in I. ALDH1L1: Aldehyde dehydrogenase 1 family member L1; AQP4: aquaporin 4; CX43: gap junction protein connexin 43; EAAT1: excitatory amino acid transporter 1; EAAT2: excitatory amino acid transporter 2; GFAP: glial fibrillary acidic protein; GLUS: glutamine synthetase; hGFAP: human-specific glial fibrillary acidic protein; hMAP2: human-specific microtube-associated protein 2; hNeuN: human-specific neuronal nuclei; hNF-L: human-specific neurofilament light chain protein; hNu: human nuclei; NeuN: neuronal nuclei; S100β: central nervous system specific protein β.

In addition to the transcription factor SOX9, mature astrocytes express several integral structural and functional proteins. Indeed, as early as 3 months after transplantation, hNu^+^ human cells within the graft expressed several cytoskeletal proteins, including vimentin (**Figure 4A**), central nervous system specific protein β (S100β) (**Figure 4B**), and GFAP (**Figure 4C**). The expression of hGFAP overlapped with pan-GFAP (**Figure 4D**). Furthermore, hNu^+^ human cells expressed several functional proteins, including enzymes such as aldehyde dehydrogenase (ALDH1L1) (**Figure 4E**) and glutamine synthetase (GLUS) (**Figure 4F**), as well as the water channel protein, aquaporin (AQP4) (**Figure 4G**). Human astrocytic processes expressed AQP4 (**Figure 4H**). Similarly, hGFAP^+^ astrocytic processes within the graft expressed the gap junction protein connexin 43 (CX43) (**Figure 4I**). Moreover, excitatory amino acid transporter 1 (EAAT1, also known as GLAST1) (**Figure 4J**) and EAAT2 (also known as GLT-1) (**Figure 4K**) were expressed in hGFAP^+^ astrocytic processes. Similarly, at 7–20 months after transplantation, differentiated human spinal astrocytes within the graft expressed skeletal and functional proteins and showed integration into the rat spinal cord (data not shown).

Human spinal astrocytes structurally interacted with the host rat spinal cord. For example, hGFAP^+^ human astrocytic processes wrapped around laminin^+^ rat blood vessels (**Figure 4L**). In addition to interacting with rat spinal cord structures, hGFAP^+^ human astrocytic processes also circled the soma of differentiated hNeuN+ human neurons (**Figure 4M**). Moreover, human-specific microtube-associated protein (hMAP2)^+^ dendrites (**Figure 4N**) and human-specific neurofilament light chain (hNF-L)^+^ axons (**Figure 4O**) from differentiated human neurons were in parallel contact with hGFAP^+^ human astrocytic processes. Finally, hGFAP^+^ human astrocytic processes projected into the host rat gray matter, where they surrounded rat interneurons (**Figure 4P**) and motor neurons (**Figure 4Q**). Altogether, these results imply that human spinal astrocytes grow and are assimilated into the rat spinal cord.

### Human cells that migrated from grafts into the rat spinal white matter are almost exclusively spinal astrocytes

**Figure 5:**
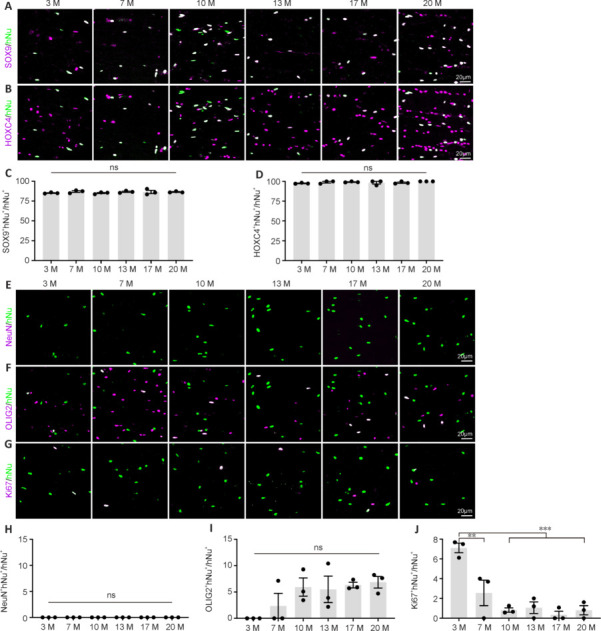
Human cells that migrated from grafts into the rat spinal white matter were almost exclusively spinal astrocytes. (A, B) Representative images of SOX9 (magenta, Alexa Fluor 647) (A), HOXC4 (magenta, Alexa Fluor 647) (B), and hNu (green, Alexa Fluor 488) staining of the white matter in horizontal sections from C5–T2 segments of the rat spinal cord at 3 M to 20 M after transplantation of hESC-derived dorsal spinal NSPCs. Numbers of SOX9^+^ cells and HOXC4^+^ cells included over 80% of cells within the white matter, and showed no obvious changes from 3 M to 20 M. (C, D) Quantification of the percentage of SOX9^+^ hNu^+^ (C) and HOXC4^+^ hNu^+^ (D) cells among hNu^+^ cells in grafts over 3–20 months. One-way analysis of variance: SOX9, *F*_(5,12)_ = 0.6175, *P* = 0.6893; HOXC4, *F*_(5,12)_ = 0.8943, *P* = 0.5150. (E–G) Representative images of NeuN (magenta, Alexa Fluor 647) (E), OLIG2 (magenta, Alexa Fluor 594) (F), Ki67 (magenta, Cy3) (G), and hNu (green, Alexa Fluor 488) staining of white matter in horizontal sections from rat spinal cord C5–T2 segments at 3 to 20 months after transplantation of hESC-derived dorsal spinal NSPCs. There were no NeuN^+^ cells from 3 M to 20 M and no OLIG2^+^ cell at 7 months. The number of OLIG2^+^ cells showed no change from 7 M to 20 M. The number of Ki67^+^ cells decreased from 3 M to 20 M. Scale bars: 20 μm in A, B, E–G. (H–J) Quantification of the percentages of NeuN^+^hNu^+^ (H), OLIG2^+^hNu^+^ (I), and Ki67^+^hNu^+^ (J) cells among hNu^+^ cells in grafts at 3–20 months. One-way analysis of variance: OLIG2, *F*_(5,12)_ = 2.689, *P* = 0.0744; Ki67, *F*_(5,12)_ = 14.83, *P* < 0.0001. Tukey’s test: Ki67, 3 M *vs*. 7 M, *P* = 0.0042; 3 M *vs.* 10 M, *P* = 0.0003; 3 M *vs.* 13 M, *P* = 0.0004; 3 M *vs.* 17 M, *P* = 0.0001; 3 M *vs.* 20 M, *P* = 0.0002. The data are presented as mean ± SEM. **P < 0.01, ***P < 0.001. hNu: Human nuclei; HOXC4: homeobox C4; M: month; NeuN: neuronal nuclei; ns: not significant; OLIG2: oligodendrocyte transcription factor 2; SOX9: SRY-box transcription factor 9.

As already described, many human astrocytes migrated into the rat spinal white matter (**Figure 2A**, **C**, and **D**), suggesting extensive migration of human cells into these areas. Indeed, human white matter cells were also positive for SOX9 (**Figure 5A**) and HOXC4 (**Figure 5B**). Quantification revealed that almost 90% of human cells that migrated to the white matter were astrocytes (**Figure 5C**), and further, were exclusively HOXC4^+^ spinal astrocytes (**Figure 5D**). In contrast, human cells that migrated into white matter were not neurons (**Figure 5E** and **H**), but a few (approximately 5%) were OLIG2^+^ oligodendroglia (**Figure 5F** and **I**). Regarding the proliferation of migrating human cells, approximately 7% expressed Ki67 at 3 months after transplantation (**Figure 5G** and **J**). This proliferation rate gradually decreased over time to less than 2% at 10–20 months after transplantation (**Figure 5G** and **J**). Taken together, the grafted human cells that migrated into the rat spinal white matter were almost all spinal astrocytes.

**Figure 6:**
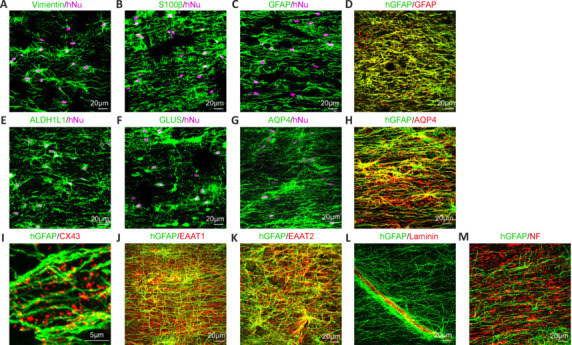
Maturation and structural integration of human spinal astrocytes in the white matter of the rat spinal cord. (A–C) Representative images of vimentin (green, Alexa Fluor 488) (A), S100β (green, Alexa Fluor 488) (B), GFAP (red, Cy3) (C), and hNu (magenta, Alexa Fluor 647) staining in the white matter of horizontal sections from C5–T2 segments of the rat spinal cord at 7 months after transplantation. (D) Representative image of GFAP (red, Cy3) and hGFAP (green, Alexa Fluor 488) staining in the white matter of horizontal sections from C5–T2 segments of the rat spinal cord at 7 months after transplantation. (E–G) Representative images of ALDH1L1 (green, Alexa Fluor 488) (E), GLUS (green, Alexa Fluor 488) (F), AQP4 (green, Alexa Fluor 488) (G), and hNu (magenta, Alexa Fluor 647) staining in the white matter of horizontal sections from C5–T2 segments of the rat spinal cord at 7 months after transplantation. (H–K) Representative images of AQP4 (red, Cy3) (H), CX43 (red, Cy3) (I), EAAT1 (red, Cy3) (J), EAAT2 (red, Cy3) (K), and hGFAP (green, Alexa Fluor 488) staining in the white matter of horizontal sections from C5–T2 segments of the rat spinal cord at 7 months after transplantation. (L, M) Representative images of laminin (red, Cy3) (L), NF (red, Cy3) (M), and hGFAP (green, Alexa Fluor 488) staining in the white matter of horizontal sections from C5–T2 segments of the rat spinal cord at 7 months after transplantation. Scale bars: 20 μm in A–H, J–M; and 5 μm in I. ALDH1L1: Aldehyde dehydrogenase 1 family member L1; AQP4: aquaporin 4; CX43: gap junction protein connexin 43; EAAT1: excitatory amino acid transporter 1; EAAT2: excitatory amino acid transporter 2; GFAP: glial fibrillary acidic protein; GLUS: glutamine synthetase; hGFAP: human-specific glial fibrillary acidic protein; hNu: human nuclei; NF: neurofilament; S100β: central nervous system specific protein β.

Similarly, as early as 7 months after transplantation, migrating hNu^+^ human cells in the white matter of rats also expressed cytoskeletal proteins, including vimentin (**Figure 6A**), S100β (**Figure 6B**), and GFAP (**Figure 6C**). Expression of hGFAP overlapped with pan-GFAP expression (**Figure 6D**). Additionally, migrating hNu^+^ human cells expressed key functional proteins, including ALDH1L1 (**Figure 6E**), GLUS (**Figure 6F**), and AQP4 (**Figure 6G**). The hGFAP^+^ human astrocytic processes expressed other essential functional proteins, including AQP4 (**Figure 6H**), CX43 (**Figure 6I**), EAAT1 (**Figure 6J**), and EAAT2 (**Figure 6K**). In terms of integration, hGFAP^+^ human astrocytic processes similarly wrapped around laminin^+^ rat blood vessels (**Figure 6L**). In addition, hGFAP^+^ human astrocytic processes were in parallel contact with rat axons (**Figure 6M**). At 10–20 months after transplantation, migrating human spinal astrocytes in the rat spinal white matter expressed similar skeletal and functional proteins and integration patterns (data not shown). These results show that the human cells that migrated from grafts into the rat spinal white matter were almost exclusively astrocytes that matured and were incorporated into the rat spinal cord structure.

### Long-distance migration, maturation, and integration of human spinal astrocytes in the rat spinal cord

**Figure 7:**
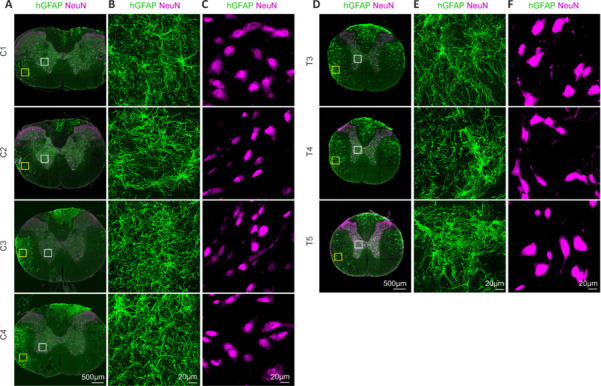
Long-distance migration of human astrocytes from grafts into rostral and caudal segments of the rat spinal cord. (A) Representative images of hGFAP (green, Alexa Fluor 488) and NeuN (magenta, Alexa Fluor 647) staining of coronal sections from C1–C4 segments of the rat spinal cord at 10 months after transplantation of hESC-derived dorsal spinal NSPCs. The number of hGFAP+ cells in the white matter decreased from C4 to C1. (B) Magnified images of the yellow boxed areas in the white matter in A. (C) Magnified images of the white boxed areas in the gray matter in A. (D) Representative images of hGFAP (green, Alexa Fluor 488) and NeuN (magenta, Alexa Fluor 647) staining of coronal sections from T3–T5 segments of the rat spinal cord at 10 months after transplantation of hESC-derived dorsal spinal NSPCs. The number of hGFAP^+^ cells in the white matter decreased from T3 to T5. (E) Magnified images of the yellow boxed areas in the white matter in D. (F) Magnified images of the white boxed areas in the gray matter in D. Scale bars: 500 μm in A, D; and 20 μm in B, C, E and F. hGFAP: Human-specific glial fibrillary acidic protein; NeuN: neuronal nuclei; NSPC: neural stem/progenitor cell.

As described, human astrocytes extensively migrated into the rat spinal white matter, beginning at 10 months after transplantation (**Figure 2A**, **C**, and **D**). We thus next determined whether further longer distance migration occurred. Rat spinal cord segments both rostral and caudal to the graft segments were sectioned into a series of coronal slices, followed by staining with hGFAP and NeuN. As expected, as early as 10 months after transplantation, hGFAP^+^ human astrocytes extended both rostrally into C4‒C1 segments (**Figure 7A**) and caudally into T3‒T5 segments (**Figure 7D**). These migrated human astrocytes were always present in the white matter, either in the lateral, ventral, or dorsal funiculus (**Figure 7A**, **B**, **D**, and **E**), but not the gray matter (**Figure 7A**, **C**, **D**, and **F**). At 13–20 months post-transplantation, hGFAP^+^ human astrocytes also progressed both rostrally into C4‒C1 segments and caudally into T3‒T5 segments (data not shown).

**Figure 8:**
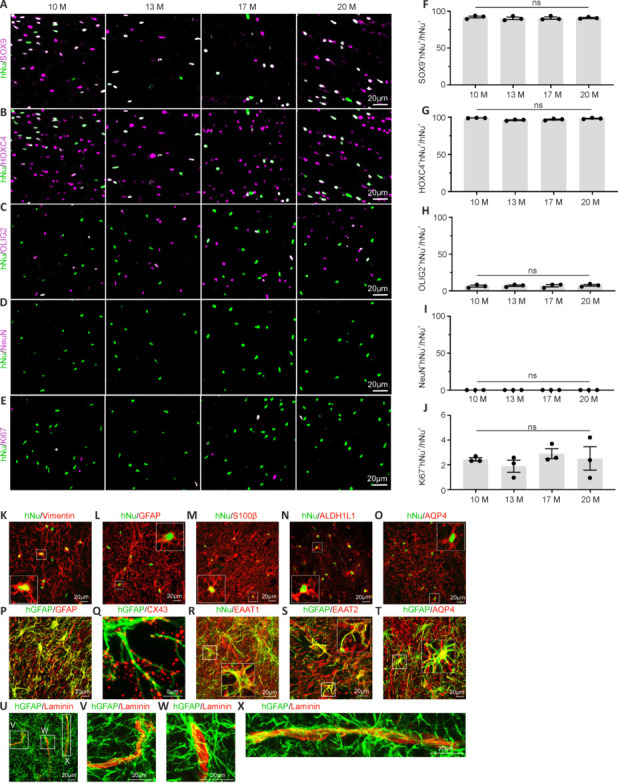
Maturation and structural integration of long-distance migrating human spinal astrocytes in the rat spinal cord. (A–E) Representative images of SOX9 (magenta, Alexa Fluor 647) (A), HOXC4 (magenta, Alexa Fluor 647) (B), OLIG2 (magenta, Alexa Fluor 594) (C), NeuN (magenta, Alexa Fluor 647) (D), Ki67 (magenta, Cy3) (E), and hNu (green, Alexa Fluor 488) staining of white matter from coronal sections of C1‒C4 and T3‒T5 segments of the rat spinal cord at 10‒20 months after transplantation. There were no NeuN^+^ cells detected within the white matter at different time points. In contrast, numbers of SOX9^+^ cells and HOXC4^+^ cells accounted for over 90% of hNu+ cells from 10 months to 20 months. There were few OLIG2^+^ cells and Ki67^+^ cells and these numbers showed no change from 10 months to 20 months. (F–J) Quantification of percentages of SOX9^+^hNu^+^ (F), HOXC4^+^hNu^+^ (G), OLIG2^+^hNu^+^ (H), NeuN^+^hNu^+^ (I), and Ki67^+^hNu^+^ (J) cells among hNu^+^ cells in the white matter of C1‒C4 and T3‒T5 segments of the rat spinal cord. One-way ANOVA: SOX9, *F*_(3,8)_ = 0.3134, *P* = 0.8155; HOXC4, *F*_(3,8)_ = 3.216, *P* = 0.0828; OLIG2, *F*_(3,8)_ = 0.1502, *P* = 0.9266; Ki67, *F*_(3,8)_ = 0.5323, *P* = 0.6728. (K–O) Representative images of vimentin (red, Cy3) (K), GFAP (red, Cy3) (L), S100β (red, Cy3) (M), ALDH1L1 (red, Cy3) (N), AQP4 (red, Cy3) (O), and hNu (green, Alexa Fluor 488) staining of white matter from coronal sections of C1‒C4 and T3‒T5 segments of the rat spinal cord. (P‒T) Representative images of GFAP (red, Cy3) (P), CX43 (red, Cy3) (Q), EAAT1 (red, Cy3) (R), EAAT2 (red, Cy3) (S), and AQP4 (red, Cy3) (T) and hGFAP (green, Alexa Fluor 488) staining of white matter in coronal sections from C1‒C4 and T3‒T5 segments of the rat spinal cord. (U‒X) Representative image of laminin (red, Cy3) and hGFAP (green, Alexa Fluor 488) staining of white matter in coronal sections from C1‒C4 and T3‒T5 segments of the rat spinal cord. The data are presented as mean ± SEM. Scale bars: 20 μm in A–E, K–P, and R–X; and 5 μm in Q. ALDH1L1: Aldehyde dehydrogenase 1 family member L1; AQP4: aquaporin 4; CX43: gap junction protein connexin 43; EAAT1: excitatory amino acid transporter 1; EAAT2: excitatory amino acid transporter 2; GFAP: glial fibrillary acidic protein; GLUS: glutamine synthetase; hGFAP: human-specific glial fibrillary acidic protein; hNu: human nuclei; HOXC4: homeobox C4; M: month; NeuN: neuronal nuclei; ns: not significant; OLIG2: oligodendrocyte transcription factor 2; SOX9: SRY-box transcription factor 9; S100β: central nervous system specific protein β.

We further characterized the fate of human cells migrating into C4–C1 and T3–T5 segments. Consistently, almost 90% of hNu^+^ human cells were positive for SOX9 (**Figure 8A** and **F**), and nearly all expressed HOXC4 (**Figure 8B** and **G**). Approximately 10% were OLIG2^+^ oligodendroglia (**Figure 8C** and **H**), while none were NeuN^+^ neurons (**Figure 8D** and **I**). Only approximately 2% of the human cells were proliferative (**Figure 8E** and **J**).

At 10 months after transplantation, these migrating human cells also expressed vimentin, GFAP, S100β, ALDH1L1, and AQP4 (**Figure 8K–O**). Again, hGFAP overlapped with pan-GFAP (**Figure 8P**). The hGFAP^+^ human astrocytic processes expressed CX43, EAAT1, EAAT2, and AQP4 (**Figure 8Q–T**). The hGFAP^+^ human astrocytic processes similarly wrapped around laminin^+^ rat blood vessels (**Figure 8U–X**). Thus, the human cells that migrated into the C4‒C1 and T3‒T5 segments were exclusively spinal astrocytes that had matured and were structurally integrated into the rat spinal cord.

## Discussion

In order to generate a human astrocytic chimeric rat spinal cord model, the first question is what type of cells should be selected for transplantation. There are two methods that could be considered: 1) directly engrafting human spinal astrocytes into naïve rats; or 2) transplanting progenitor cells which can differentiate into human spinal astrocytes *in vivo*. The first method needs *in vitro* production of human spinal astrocytes, which takes more time and effort than the acquisition of progenitor cells (Haidet-Phillips et al., 2014). Therefore, we chose to use the second method, in which *in vitro* acquired progenitor cells are used to produce human spinal astrocytes *in vivo*. This method enabled us to systematically study the genesis and function of human spinal astrocytes. Thus, human dorsal spinal NSPCs generated from hESCs were used to generate a human astrocytic chimeric rat spinal cord model. Previous studies have differentiated human spinal astrocytes from hESCs and hiPSCs (Krencik et al., 2011; Roybon et al., 2013; Holmqvist et al., 2015; Taga et al., 2019). However, RA was used to pattern the neuroepithelia towards a spinal cord identity, with HOXB4 used as a spinal marker to characterize differentiated NSPCs, similar to our initial studies (Chen et al., 2015; Qian et al., 2017). RA leads to activation of only HOX1–HOX5 chromatin domains (Mazzoni et al., 2013; Lippmann et al., 2015), while HOXB4 is not specific to the spinal cord, as its expression is also present in the hindbrain (Philippidou and Dasen, 2013; Lippmann et al., 2015). In contrast, activation of WNT signaling induces expression of the CDX2 transcription factor, which binds to the HOX1–HOX9 chromatin domains, leading to specification of an authentic spinal identity (Mazzoni et al., 2013; Lippmann et al., 2015). Indeed, our differentiated neuroepithelia almost uniformly expressed CDX2 and spine-specific HOX genes, including HOXC4, HOXB8, and HOXC9, consistent with a study in which human spinal cord neural stem cells (NSCs) were generated using CHIR to activate WNT signaling (Kumamaru et al., 2018). Moreover, when transplanted into the rat spinal cord, our surviving and differentiating cells were also almost exclusively positive for HOXC4, which is expressed in adult human spinal cord cells, including astrocytes (Yadav et al., 2023; Zhang et al., 2024). Accordingly, the previously differentiated so-called spinal astrocytes identified by others (Krencik et al., 2011; Roybon et al., 2013; Holmqvist et al., 2015; Taga et al., 2019) and us using RA (Chen et al., 2015; Qian et al., 2017) might be a mixed hindbrain and most rostral population of spinal astrocytes. In contrast, in the present study, our differentiated astrocytes were cervical and thoracic in positional identity, supporting the finding that they are a true spinal chimeric model.

Our human dorsal spinal NSPCs were generated from hESCs after 3 weeks and then directly transplanted into the rat spinal cord without further differentiation into mature astrocytes *in vitro*, as described previously for hindbrain astrocytes (Haidet-Phillips et al., 2014). This approach was used because *in vitro* differentiation of mature human astrocytes is time-consuming. The earliest protocol took at least 180 days for the maturation of both cerebral and hindbrain astrocytes (Krencik et al., 2011). Even with modified protocols in which transcription factors (including SOX9 and NF1A/B) are overexpressed for rapid generation of human astrocytes, it still takes at least 2–3 months for the astocytes to mature (Canals et al., 2018; Li et al., 2018; Tchieu et al., 2019; Soubannier et al., 2022; Hosseini et al., 2024). Difficulties in increasing the maturation of differentiated human central nervous system cells *in vitro* have also been encountered in neural organoids (Revah et al., 2022). However, the latest research has shown that once human cortical organoids are transplanted into rodent brains, they accelerate to maturation and exhibit morphological and synaptic characteristics that are more complex than their *in vitro* counterparts (Revah et al., 2022). Thus, we transplanted dorsal spinal NSPCs rather than mature astrocytes to save time. Indeed, engrafted human dorsal spinal NSPCs differentiated into high-purity spinal astrocytes as early as 3 months after engraftment. This is consistent with our previous studies in which hESCs and hiPSC-derived hindbrain NSPCs generated the highest number of astrocytes after engraftment into the mouse spinal cord (Chen et al., 2015; Qian et al., 2017). Our engrafted human spinal astroglia expressed many structural and functional proteins, including vimentin, GFAP, S100β, ALDH1L1, GLUS, AQP4, EAAT1, EAAT2, and CX43. In the adult human spinal cord, these genes are highly expressed in astrocytes, as determined by single-nucleus RNA sequencing (Yadav et al., 2023; Zhang et al., 2024). Thus, the engrafted human spinal astrocytes may have matured. In addition, our spinal astrocytes migrated extensively into the white matter for long distances both rostrally and caudally, and interacted structurally with both human and rat neurons, dendrites and axons, forming a blood–spinal cord barrier. This is similar to the migration and integration patterns of human forebrain (Lu et al., 2017; Lien et al., 2019) and hindbrain astrocytes (Chen et al., 2015; Qian et al., 2017) in the rodent spinal cord. Taken together, to establish a human spinal astrocytic chimeric model, it appears sufficient to directly transplant dorsal spinal NSPCs instead of mature astrocytes.

There are several aspects that deserve attention. First, regarding the differentiation of transplanted neural progenitors, the dominant astrocytic fate observed in our study could be attributed to both environmental cues and the intrinsic properties of graft cells (Beyer et al., 2019). As reported in previous studies, NSCs preferentially adopt glial phenotypes when transplanted into the adult spinal cord (Chen et al., 2015; O’Shea et al., 2022). This differentiation preference is partially due to a lack of neurogenic cues in the adult spinal cord, which needs relevant molecules and cells (Horner et al., 2000; Park et al., 2003). Moreover, intrinsic features should be considered when exploring the variability in differentiated cell fates. In general, human NSCs are more potent at producing neuronal descendants than their rodent counterparts (Iwanami et al., 2005). Nevertheless, one study in which hESC/iPSC-derived spinal NSPCs were transplanted into intact spinal cords reported that approximately 68.5% of human cells expressed the astrocyte marker, GFAP, at 9 months post-graft (Chen et al., 2015). As the differentiation of astrocytes is slower than neurogenesis (Guillaume et al., 2006), we assumed that the percentage of human cells taking on a glial lineage would increase with increasing time post-graft. Additionally, the majority of astrocytes originate from the dorsal spinal cord during development, whereas NSCs located in the ventral spinal cord differentiate into oligodendrocytes (Li et al., 2023). The dorsal NSCs used in our study were more likely to produce astrocytes than oligodendrocytes. Overall, we believe that the high differentiation ratio of astrocytes in our studies is not only consistent with previous reports of a constitutively unfavorable environment in the adult spinal cord but also fits the intrinsic characteristics of dorsal NSCs in the human spinal cord. Female rats were selected for their shorter and straighter urethras, which facilitate manual urine drainage in case of transient spinal dysfunction (including urine retention) after transplantation. Fortunately, no rats exhibited such symptoms post-grafting.

Extensive migration of hESC-derived astrocytes was present in the host white matter instead of gray matter, which involves more than five spinal segments in both the rostral and caudal directions along the longitudinal spinal axis. This finding confirmed our previous report that hESC/iPSC-derived NSCs generated migrating astrocytes that were distributed exclusively in the white matter and with a distance as long as 9 mm in the rodent spinal cord (Chen et al., 2015). Similar migrating behaviors of differentiated astrocytes have been widely observed, independent of the species and tissues of donor cells as well as the condition of the host spinal cord (intact or injured) (Han et al., 2004; Lu et al., 2017; Lien et al., 2019). However, the mechanisms mediating this specific migration pattern remain elusive. The preference of graft-derived astrocytes for migrating into white matter could be attributed to the distinct microenvironment of white matter compared with gray matter. For example, white matter is defined by a different composition of extracellular matrix components and signaling molecules that may enhance astrocyte migration and integration (Werkman et al., 2021). Molecules such as the chemokine CXCL1 (Tsai et al., 2002), platelet-derived growth factor (Calver et al., 1998), and netrin-1 (Jarjour et al., 2003; Tsai et al., 2003) are reported to be either chemorepellents or chemoattractants that guide glial migration in the spinal cord. Moreover, studies have indicated that myelin itself can act as a chemoattractant for various cell types, including astrocytes and oligodendrocyte progenitor cells (Wang et al., 2011; Seiler et al., 2024). However, whether these and other molecules modulate graft-derived astrocyte migration warrants further research. Our results indicate that the human astrocytes extensively migrated into the rat spinal white matter, thereby establishing a chimeric spinal model. This model can be used to explore the behaviors of human astrocytes normally or under pathological conditions such as SCI.

Accumulating evidence has identified beneficial effects of graft-derived human astrocytes following SCI (Zheng and Wang, 2022). Accordingly, the transplantation of human astrocytes can promote neuroprotection, enhance axonal regeneration, and improve functional recovery post-injury (Liang et al., 2024). For example, grafted astrocytes derived from human glial restricted progenitors can create a permissive environment for host axons, facilitating their growth and integration into the spinal cord circuitry (Haas and Fischer, 2013). Moreover, when transplanted into the contused spinal cord, human glial restricted progenitors and differentiated human astrocytes can reduce the size of the lesion cavity, attenuate the development of glial and fibrotic scars, and promote the regrowth of serotonergic fibers, indicating both neuroprotective and neuroregenerational benefits (Jin et al., 2011). As a consequence, sensory and bladder functions recover significantly despite the lack of effects on motor function (Jin et al., 2011). The therapeutic potential of astrocyte transplantation is further highlighted by studies using human astrocytes derived from hESCs or hiPSCs, which identified an ability of these cells to protect neurons from excitotoxicity and support neuronal functions via the regulation of neurotransmitter levels (Deligne et al., 2015; Lien et al., 2019). However, in all of these studies, human astrocytes were transplanted post-injury, with the manifested functionality possibly confounded by transplantation procedures. To better mimic the injury-induced responses of human astrocytes, we decided to first establish a chimeric spinal model in which human astrocytes steadily reside, and thereafter induce injury. In this study, we completed the first step of establishing a chimeric spinal model.

A key limitation of our current study is that the functional properties of human spinal astrocytes were not examined *in vivo*. However, we are now exploring the role of human spinal astrocytes in SCI. After engraftment of hESC-derived dorsal spinal NSPCs into adult nude rat spinal cords, injury to the transplant site is implemented to first observe the reactions of human spinal astrocytes, followed by manipulations to determine their contributions to functional recovery after SCI. These experiments are in progress. Female rats were selected for their shorter and straighter urethras, which facilitate manual urine drainage in case of transient spinal dysfunction, including urine retention, after transplantation. Therefore, there may be sex bias in the experiment.

In summary, we have successfully developed a human astrocytic chimeric rat spinal cord model by transplanting hESC-derived dorsal spinal NSPCs into the spinal cord of adult naïve nude rats. This model provides a valuable tool for further investigating the responses of human spinal astrocytes in various spinal cord diseases and lays the groundwork for future efforts to explore the potential use of human spinal astrocytes for treating spinal diseases.

## Data Availability

*All the data reported in this paper will be shared by the lead contact upon request. This paper does not report the original code. Any additional information needed to reanalyze the data reported in this paper is available from the lead contact upon request*.
